# Open conformation of hERG channel turrets revealed by a specific scorpion toxin BmKKx2

**DOI:** 10.1186/2045-3701-4-18

**Published:** 2014-04-11

**Authors:** You-Tian Hu, Jun Hu, Tian Li, Jing-Jing Wei, Jing Feng, Yi-Mei Du, Zhi-Jian Cao, Wen-Xin Li, Ying-Liang Wu

**Affiliations:** 1 State Key Laboratory of Virology, College of Life Sciences, Wuhan University, Wuhan 430072, China; 2 Research Center of Ion Channelopathy, Institute of Cardiovascular Diseases, Union Hospital, Tongji Medical College, Huazhong University of Science and Technology, Wuhan 430022, China

**Keywords:** Scorpion toxin, BmKKx2, hERG channel, Turret, Pore region, Molecular mechanism

## Abstract

**Background:**

The human ether-a-go-go-related gene potassium channel (hERG) has an unusual long turret, whose role in recognizing scorpion toxins remains controversial. Here, BmKKx2, the first specific blocker of hERG channel derived from scorpion *Mesobuthus martensii*, was identified and the turret role of hERG channel was re-investigated using BmKKx2 as a molecular probe.

**Results:**

BmKKx2 was found to block hERG channel with an IC_50_ of 6.7 ± 1.7 nM and share similar functional surface with the known hERG channel inhibitor BeKm-1. The alanine-scanning mutagenesis data indicate that different residue substitutions on hERG channel by alanine decreased the affinities of toxin BmKKx2 by about 10-fold compared with that of wild-type hERG channel, which reveals that channel turrets play a secondary role in toxin binding. Different from channel turret, the pore region of hERG channel was found to exert the conserved and essential function for toxin binding because the mutant hERG-S631A channel remarkably decreased toxin BmKKx2 affinity by about 104-fold.

**Conclusions:**

Our results not only revealed that channel turrets of hERG channel formed an open conformation in scorpion toxin binding, but also enriched the diversity of structure-function relationships among the different potassium channel turrets.

## Background

Potassium channels mediate K^+^ efflux and play various pharmacological functions [1]. Structurally, the potassium channels present various topologies, such as inwardly rectifying two-transmembrane potassium channels (Kir), calcium-activated potassium channels with six or seven transmembrane segments (KCa) and voltage-gated potassium channels with six transmembrane segments (Kv) [2]. Due to the channel structure complexity and technique challenge, there are a few crystal structures of eukaryotic potassium channels, such as Kv1.2, Kir2.2 and Kir3.2 [3-5]. Comparison of these crystal structures reveals essential conformation differences in the extracellular pore entryway that includes turret and filter regions. In recent years, other structural features of channel turrets were also revealed by the specific scorpion toxins as the molecular probes. For example, the 10-residue turrets of Kv1.2 channel is induced to form an open state conformation and not affect toxin binding activity when it is bound by scorpion toxin maurotoxin [6]. Different from the open state conformation of Kv1.2 channel turret, the 17-residue turrets of small conductance KCa3 (SKCa3) channel form a “peptide screener” compact conformation which selectively controls scorpion toxin binding [7]. More interestingly, the 19-residue turret of large conductance KCa (BKCa) channel switches from an open state conformation for scorpion toxin charybdotoxin (ChTX) binding to a compact “helmet” conformation for toxin ChTX insensitivity when the channel turret interacts with its auxiliary β4 subunits [8]. Besides above progress of structure and function relationships of different potassium channel turrets, characterization of more potassium channel extracellular pore entryways remains a challenge nowadays due to the difficulties in determining the crystal structures of additional potassium channels.

Among all the superfamily members of potassium channels, hERG (human ether-a-go-go-related gene) potassium channel has an unusual longer turret containing 40 amino acid residues [9]. Using the scorpion toxin BeKm-1 as a molecular probe, different conformational states in the turret of hERG channel have been reported. Through the computational simulation technique, our group ever predicted that hERG channel vestibule presented an open conformation mainly in a decentralized ‘petunia’ shape and turrets were far from the bound toxin BeKm-1 [10], while another model without a ‘petunia’ shape was also proposed by other group, in which channel turrets directly interacted with the bound BeKm-1 [11]. Thus, these controversial structural-functional features still need to be investigated using scorpion toxins as a molecular probe. In this work, we revisit the conformational state of hERG channel turrets by using a specific scorpion toxin BmKKx2, a novel toxin from Scorpion *Mesobuthus martensii* with similar structural and functional features as BeKm-1 [12]. Through alanine-scanning mutagenesis and pharmacological experiments, hERG channel pore region instead of turrets was mainly responsible for toxin BmKKx2 binding, supporting our previous inference that the hERG channel turret adopts an open conformation. Taken together, these findings provide new insights into the role of channel turret in toxin recognition and might help to better understand the structure-function relationship of hERG channel.

## Results

### Expression and purification of BmKKx2

**Figure 1:**
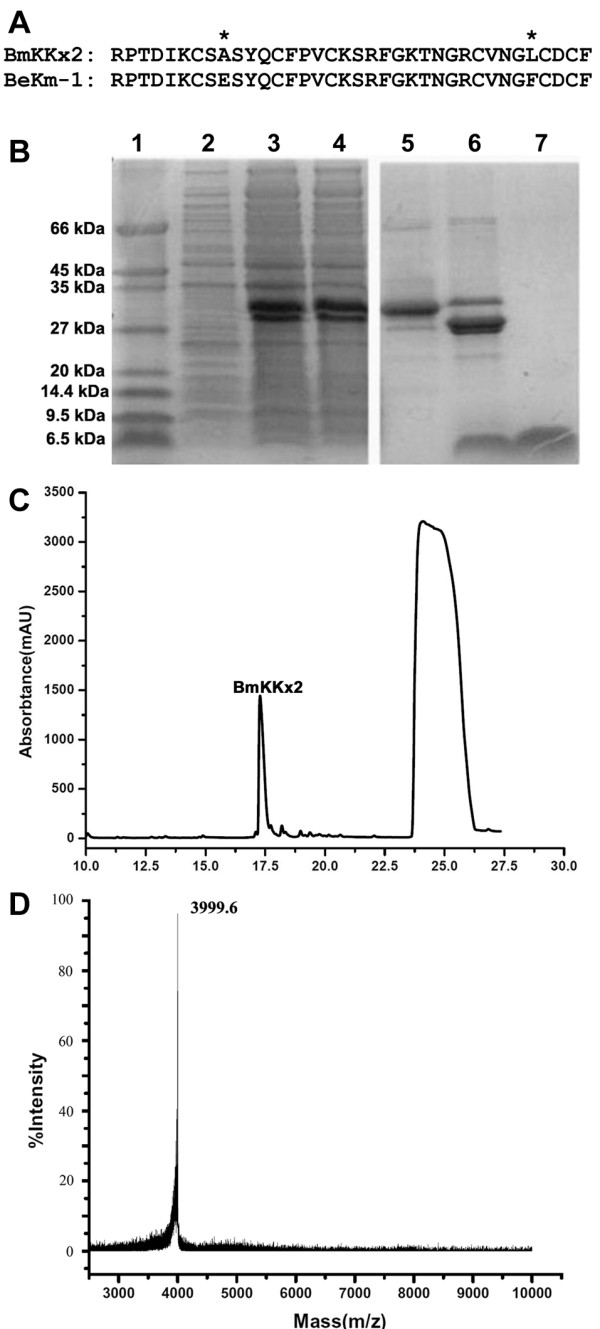
**Purification and characterization of toxin BmKKx2. (A)** Amino acid sequence alignment for BmKKx2 and BeKm-1. The differential residues were marked with asterisks. **(B)** Tricine-SDS-PAGE analysis of purified toxin BmKKx2. Lane 1, molecular mass markers; lane 2, non-induced cell-free extract of *E. coli* carrying pGEX-6p-1-BmKKx2; Lane 3, total cell-free extract of *E. coli* carrying pGEX-6p-1-BmKKx2 induced with IPTG for 4 h; lane 4, the purified GST-BmKKx2 protein after affinity chromatography; lane 5, the purified GST fusion protein after desalting and concentration; lane 6, the cleaved fusion protein via enterokinase; lane 7, the purified toxin BmKKx2 through reversed phase HPLC. **(C)** HPLC profile of the GST-BmKKx2 fusion protein cleaved through enterokinase. **(D)** Mass spectrum of BmKKx2 peptide measured on MALDI-TOF-MS.

As shown in Figure 1A, there are only two differential residues between BmKKx2 and BeKm-1, which suggested that BmKKx2 might also act as a hERG channel blocker [12,13]. To evaluate the pharmacological feature of BmKKx2, we obtained recombinant BmKKx2 as described previously [7,10].

The GST-BmKKx2, a fusion protein of 30 kDa size, was purified and split into two products, GST of 26 kDa and BmKKx2 of 4.1 kDa (Figure 1B). The mixture was further separated by HPLC, and two product peaks were obtained (Figure 1C). The component eluting at about 18 min corresponding to BmKKx2 was collected manually and lyophilized. By the matrix-assisted-laser-desorption/ionization time-of-flight mass spectrometry (MALDI-TOF-MS), the molecular weight of recombinant BmKKx2 was 3999.6 (Figure 1D). Considered the loss of 6 Da by three pairs of the conserved disulfide bridges, the determined molecular weight was in good agreement with the theoretical molecular weight of 4005.6 Da calculated by the ExPASy Protemics Server ( http://us.expasy.org/tools/protparam.html).

### BmKKx2 appears to be a selective hERG channel blocker

**Figure 2:**
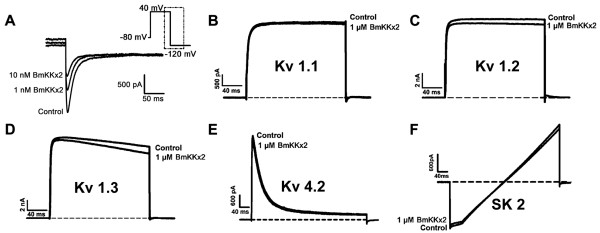
**Pharmacological profiles of BmKKx2 on cloned potassium channels. (A)** Current traces in the absence (control) or presence 1 nM and 10 nM BmKKx2 on hERG channels. **(B-F)** Current traces in the absence (control) or presence 1 μM BmKKx2 on Kv1.1, Kv1.2, Kv1.3, Kv 4.2 and SKCa2 channels.

The recognition process between toxin peptides and potassium channels remains interesting and complicated. A slight change of toxin peptides, even a single residue modified, may induce a distinct recognizing pattern [14-16]. Considering BmKKx2 as a new member of γ-K^+^-channel toxin subfamily, the blocking activity was evaluated on HEK293 cells that transiently expressed hERG channel. As shown in Figure 2A, BmKKx2 could effectively block hERG channel with an IC_50_ of 6.7 ± 1.7 nM, which was comparable to that of BeKm-1, an effective blocker of hERG channel [12]. Furthermore, we also investigated its pharmacological effects on other potassium channels, including Kv1.1, Kv1.2, Kv1.3, Kv4.2 and SKCa2. The results revealed that BmKKx2 had less effect on these potassium channels even at a concentration of 1 μM (Figure 2B-F). These data revealed BmKKx2 to be the first hERG channel selective inhibitor derived from scorpion *Mesobuthus martensii*, and making BmKKx2 a potential molecular probe of hERG channel.

**Figure 3:**
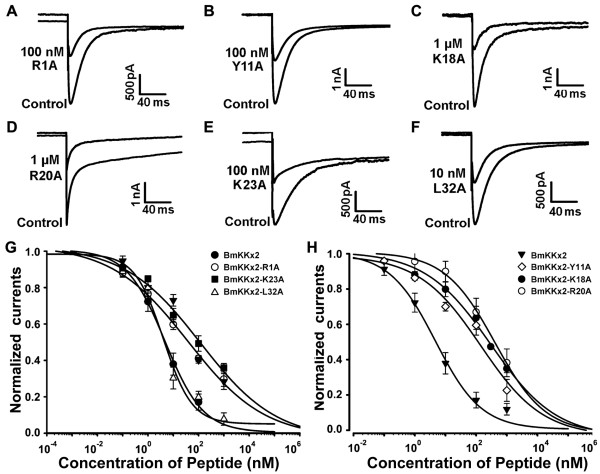
**Effects of the BmKKx2 mutants on hERG channel currents. (A-F)** Representative current traces of hERG channel showing the blocking activities of BmKKx2 mutants. **(G-H)** Dose-dependence curves of BmKKx2 mutants on hERG channels.

Besides the selectivity of toxin BmKKx2, its functional surface was also investigated. Six candidate residues (Arg1, Tyr11, Lys18, Arg20, Lys23 and Leu32) were selected and respectively mutated to alanine according to the functional map of molecular surface of BeKm-1 [17], Compared with the wild-type BmKKx2, all mutants showed less significant changes in the secondary structure through CD spectra, indicating these mutants adopted the same overall structural topology with BmKKx2 (see Additional file 1: Figure S1). Next, the blocking activities of these mutant peptides were tested on hERG channel (Figure 3) and all these results were listed in Table 1. Compared with wild-type BmKKx2, BmKKx2-K18A and BmKKx2-R20A exhibited dramatic drop of affinity on hERG channels for 56- and 61-fold, respectively (Figure 3C,D and H). Furthermore, Tyr11 and Lys23 of BmKKx2 were found moderately important for binding (Figure 3B,E,G and H), while Arg1 and Leu32 exhibited less effect on toxin blocking activities (Figure 3A, F and G). All these data revealed that BmKKx2 mainly used Tyr11, Lys18, Arg20 and Lys23 as the functional residues to recognize hERG channel, which was similar to the binding surface of toxin BeKm-1.

**Table 1:** The binding affinities of wild-type BmKKx2 and its mutants towards hERG channel

**BmKKx2 mutant**	**IC50**^***a***^	***n***^***b***^	**IC**_**50(mut)**_**/IC**_**50(wt)**_
	***nM***		
Wild type	26.7 ± 1.7	6	1.0
R1A	238.3 ± 11.2	4	8.1
Y11A	131.0 ± 35.2	4	27.9
K18A	376.6 ± 62.0	6	80.1
R20A	409.6 ± 56.9	5	87.1
F21A	863.4 ± 73.4	5	183.7
K23A	123.2 ± 56.3	4	26.2
R27A	262.7 ± 25.9	4	13.3
F32A	24.1 ± 1.9	4	0.9

^*a*^IC_50_ value(half-maximum inhibition concentration) represents as Mean ± Standard Error.

^*b*^number of independent experiments.

### Less effect of channel turret on BmKKx2 binding

**Figure 4:**
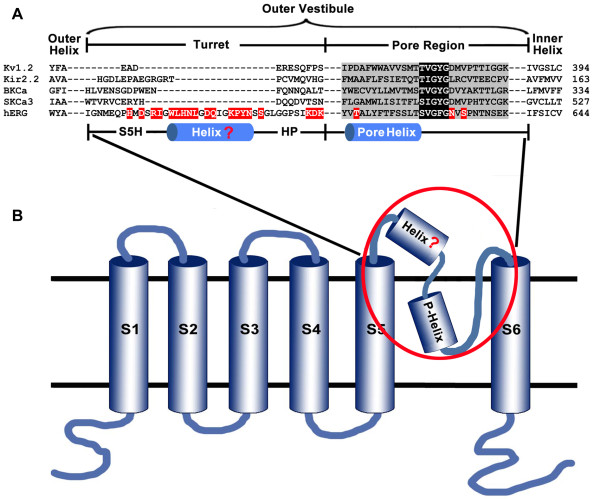
**Alignment of the outer vestibules of hERG channel and other potassium channels. (A)** Primary sequence alignment for hERG channel and other potassium channels. Black shading shows the selectively filter, whereas gray background indicates pore region of potassium channels, respectively. Red letters highlight the residues selected for alanine-scanning mutagenesis. These residues are H578, D580, R582, I583, W585, L586, H587, N588, L589, D591, Q592, K595, P596, Y597, N598, S600, K608, D609, K610, T613, N629 and S631. **(B)** Diagram of hERG channel subunit containing six transmembrane domains and possible helix structure in channel turret.

With the help of specific scorpion toxin BmKKx2 as a molecular probe, the extracellular pore entryway role of hERG channel was revisited in this work. Different from the classic potassium channels, hERG channel has an unusual longer turret containing 40 amino acid residues (Figure 4), whose role remains confused in the toxin-channel interaction [10,11]. The middle part of turret (W585 ~ Y597) was found to form a helix conformation in the hydrophobic environment but predominantly present a random coil structure in aqueous solution [18]. This possible helix divided the long turret into three regions: S5-helix linker region (S5H), helix region, helix-pore helix linker region (HP). Here, the roles of these three regions were respectively investigated during the toxin BmKKx2-hERG channel interaction.

**Figure 5:**
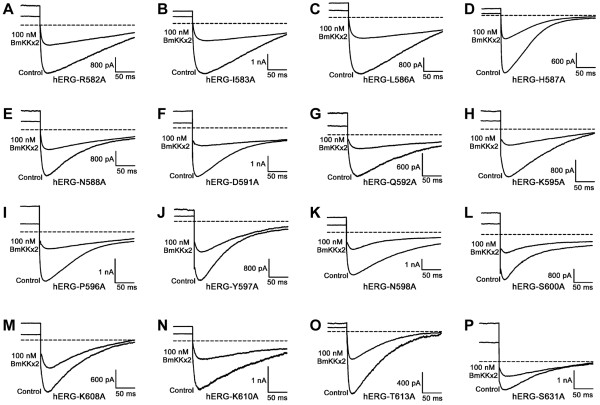
**Effects of the turret and pore region residues of hERG channel on BmKKx2 binding affinity. (A-P)** Representative current traces of hERG channel mutants showing current blocks by BmKKx2. **(A)** hERG-R582A, **(B)** hERG-I583A, **(C)** hERG-L586A, **(D)** hERG-H587A, **(E)** hERG-N588A, **(F)** hERG-D591A, **(G)** hERG-Q592A, **(H)** hERG-K595A, **(I)** hERG-P596A, **(J)** hERG-Y597A, **(K)** hERG-N598A, **(L)** hERG-S600A, **(M)** hERG-K608A, **(N)** hERG-K610A, **(O)** hERG-T613A and **(P)** hERG-S631A.

**Figure 6:**
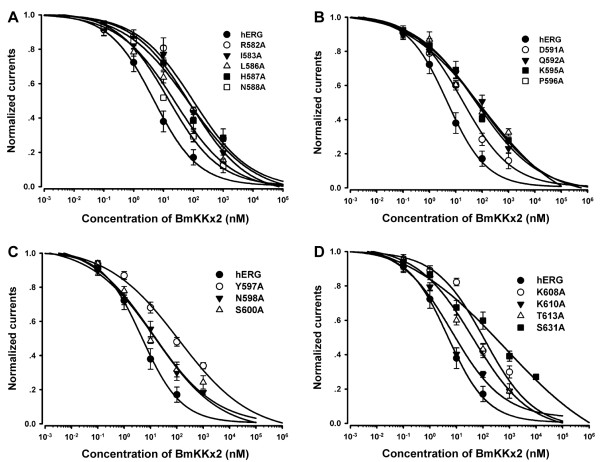
**Dose-dependent inhibition of hERG channel mutants by BmKKx2. (A)** hERG-R582A, hERG-I583A, hERG-L586A, hERG-H587A and hERG-N588A. **(B)** hERG-D591A, hERG-Q592A, hERG-K595A and hERG-P596A. **(C)** hERG-Y597A, hERG-N598A and hERG-S600A. **(D)** hERG-K608A, hERG-K610A, hERG-T613A and hERG-S631A. The IC_50_ values are listed in Table 2.

Firstly, the involvement of S5H region (I571 ~ G584) was identified using the alanine-scanning strategy. Since the N-terminal of S5H region, near the channel S5 transmembrane helix (outer helix in Figure 4), is far from the central pore and likely plays an unimportant role in toxin binding, four residues (His578, Asp580, Arg582 and Ile583) in the C-terminal of S5H region were selected and replaced by alanine, individually. It was found that the mutant channel hERG-H578A did not express and hERG-D580A mediated too small currents, making them inappropriate for evaluating their sensitivity towards BmKKx2. For the mutant channels hERG-R582A and hERG-I583A, about 70% of the currents were inhibited by 100 nM BmKKx2 (Figure 5A,B). As shown in Table 2, the dose-dependent inhibition of mutant channels by BmKKx2 indicated that the IC_50_ values of toxin BmKKx2 on hERG-R582A and hERG-I583A channels were 76.5 and 87.5 nM, respectively (Figure 6A). In comparison with the wild-type hERG channel, two mutant hERG-R582A and hERG-I583A channels respectively decreased the BmKKx2 sensitivity by 11.4- and 13.2-fold. These data showed that S5H region had less effect on the toxin BmKKx2 binding, which was in line with the investigation by the homologous scorpion toxin BeKm-1 [17].

**Table 2:** The binding affinities of toxin BmKKx2 towards wild-type and mutant hERG channel

**hERG mutant**	**IC50**^***a***^	**n**^***b***^	**IC**_**50(mut)**_**/IC**_**50(wt)**_
	nM		
Wild Type	6.7 ± 1.7	7	1.0
H578A	N.E^*c*^	5	-^*e*^
D580A	L.E^*d*^	5	-
R582A	76.5 ± 32.6	4	11.4
I583A	87.5 ± 16.8	4	13.1
W585A	N.E	6	-
L586A	24.3 ± 8.4	4	3.6
H587A	59.7 ± 31.7	5	8.9
N588A	14.5 ± 4.3	4	2.2
L589A	L.E	6	-
D591A	19.6 ± 1.7	4	2.9
Q592A	89.5 ± 39.8	5	13.4
K595A	65.2 ± 19.1	5	9.7
P596A	60.8 ± 23.4	4	9.1
Y597A	118.2 ± 26.7	4	17.6
N598A	15.2 ± 4.8	4	2.3
S600A	12.1 ± 3.5	4	1.8
K608A	116.1 ± 29.2	5	17.3
D609A	L.E.	8	-
K610A	6.7 ± 5.1	5	1.0
T613A	42.6 ± 17.1	5	6.4
N629A	L.E	7	-
S631A	698.0 ± 64.4	5	104.2

^*a*^IC_50_ value(half-maximum inhibition concentration) represents as Mean ± Standard Error.

^*b*^number of independent experiments.

^*c*^no expression, can’t detect fluorescent flag in all transfected HEK 293 T cells.

^*d*^low expression, with detectable fluorescent flag but can only elicit weak current in all transfected HEK 293 cells.

^*e*^- means the lack of IC_50_ value of mutants.

Secondly, we continued to investigate the effect of the controversial helix region in channel turrets on toxin BeKm-1 binding [10,11]. Except no expression of hERG-W585A and lower expression level of hERG-L589A, other eight mutant channels (hERG-L586A, hERG-H587A, hERG-N588A, hERG-D591A, hERG-Q592A, hERG-K595A, hERG-P596A and hERG-Y597A) exhibited the similar properties to that of wild-type hERG channels, and their effects on toxin BmKKx2 binding were determined (Figure 5C-J). The corresponding IC_50_ values of toxin BmKKx2 were 24.3, 59.7, 14.5, 19.6, 89.5, 65.2, 60.8 and 118.2 nM for hERG-L586A, hERG-H587A, hERG-N588A, hERG-D591A, hERG-Q592A, hERG-K595A, hERG-P596A and hERG-Y597A, respectively (Figure 6A-C and Table 2). Overall, there were drops of no more than 20-fold toxin sensitivity towards eight mutant hERG channels, which indicated that the helix region in channel turrets did not play an important role in scorpion toxin recognition.

Finally, the influence of the HP linker in channel turret in BmKKx2 recognition was also studied. Six mutant channels were constructed, and mutant hERG-D609A channel could not be investigated due to the negligibly small currents. Other five mutant channels (hERG-N598A, hERG-S600A, hERG-K608A, hERG-K610A and hERG-T613A) were able to mediate potassium currents with comparable amplitude with wild-type hERG channel, and also were effectively blocked by 100 nM toxin BmKKx2 (Figure 5K-O). The IC_50_ values of toxin BmKKx2 for hERG-N598A, hERG-S600A, hERG-K608A, hERG-K610A and hERG-T613A were 15.2, 12.1, 116.1, 6.7 and 42.6 nM, respectively (Figure 6C and D). Similar to the previous two S5H and helix regions of the channel turret (Table 2), these experimental data of HP linker residues indicated that it also played a secondary role in the toxin BmKKx2 binding.

### Critical role of channel pore region in BmKKx2 binding

The interaction between animal toxin blockers and potassium channels indicated that the channel pore region played a critical role in toxin recognition [19,20]. In this work, two residues Asn629 and Ser631 near the channel selectivity filter were selected and substituted by alanine residue, respectively. Since the lower expression of the mutant channel hERG-N629A, only mutant hERG-S631A channel was investigated for toxin BmKKx2 recognition. As shown in Figure 5P, mutant hERG-S631A channel currents could be inhibited by 100 nM toxin BmKKx2, and its IC_50_ of toxin BmKKx2 was 698 ± 64.4 nM, making hERG-S631A approximately 104-fold less sensitive to BmKKx2 than wild-type hERG channel (Figure 6D). The remarkable effect of channel Ser631 on toxin binding confirmed that the channel pore region was greatly involved in toxin binding.

## Discussion

With about 100 members, the potassium channels present the diverse structure-function relationships. The extracellular pore entryway including turrets and filter regions is the critical domain responsible for the inactivation, animal toxin recognition and interactions between channel subunits. Due to the difficulties in solving the crystal structures of potassium channels, the scorpion toxin blockers are useful molecular probes to characterize the structure-function relationships of the different channel extracellular pore entryways [6-8,20].

In this work, we revisited the hERG channel turret with an unusual longer turret containing 40 amino acid residues (Figure 4), whose role was ever intensively investigated in the scorpion toxin binding [10,11,17]. However, there were two different inferences on the orientation of hERG channel turrets: one is the helix domain of hERG channel turrets responsible for scorpion toxin binding through the cysteine-scanning mutagenesis and computational modeling [11], the other is the turret helix domain far from the bound scorpion toxin through the computational modeling [10]. Unexpectedly, the cysteine substitutions of hERG channel turrets at different positions were found to have high-impact, intermediate-impact or low-impact on channel functions and affect scorpion toxin binding due to the potential formation of the disulfide bonds [11,22]. In order to avoid the effect of disulfide bonds on scorpion toxin binding, the alanine-scanning mutagenesis was used to re-investigate the role of hERG channel turret, especially the controversial turret helix domain, in the scorpion toxin binding.

### Conserved functional surface of new toxin BmKKx2

**Figure 7:**
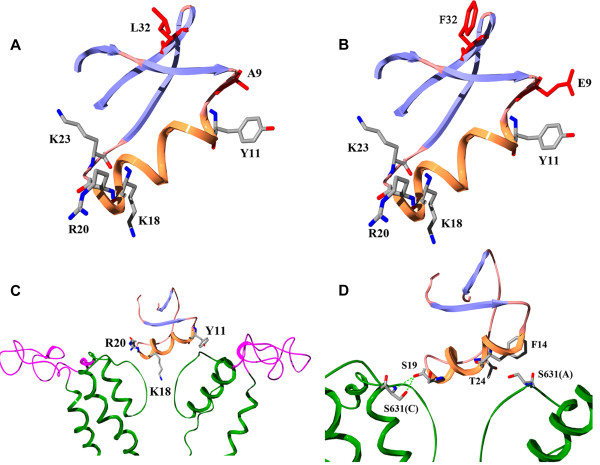
**Stuctural model and analysis of toxin BmKKx2 and its complex with hERG channel. (A-B)** The distribution of functional residues and differential residues between BmKKx2 and BeKm-1. **(A)** BmKKx2, **(B)** BeKm-1. **(C)** The spatial orientation of hERG channel turrets and toxin BmKKx2 in their complex structural. **(D)** Interaction details between hERG channel Ser 631 residues and toxin residues.

In order to explore the functional role of hERG channel turrets in scorpion toxin recognition, a homologous scorpion toxin BmKKx2 specific for hERG channel was identified with an IC_50_ of 6.7 ± 1.7 nM (Figure 2). Furthermore, the structure-function relationship of toxin BmKKx2 indicated that it shared the same critical residues (Tyr11, Lys18, Arg20 and Lys23) as those of nearly identical scorpion toxin BeKm-1 (Figures 3 and 7A-B, and Table 1) [12]. These results demonstrated that toxin BmKKx2 was also a useful molecule probe to explore the functional role of hERG channel turrets in toxin recognition.

### Open conformation of hERG channel turrets in toxin BmKKx2 recognition

As the role of channel turrets in scorpion toxin binding, the controversial issue was whether the helix domain interacted with the bound toxin (Figure 4) [10,11] although this helix domain actually did not exist due to locating above the extracellular pore entryway in the aqueous solution instead of the hydrophobic environment [9]. In previous cysteine-scanning experiments [11], total 29 mutant channels were investigated while their cysteine substitutions had intermediate-impact or low-impact on the channel function [22]. It was found that the substitution of Gln592 by the cysteine in the helix domain significantly decreased the affinity of toxin BeKm-1 by about 54-fold, and the remaining 28 turret residue substitutions including three S5H, helix and HP regions (Figure 4) decreased toxin BeKm-1 affinities by less than 10-fold when compared with the that of wild-type hERG channel. These results supported the inference that helix domain of channel turrets would interact with toxin BeKm-1. In our work, 15 channel turret residues were successfully assessed through the alanine-scanning mutagenesis (Figures 5 and 6, and Table 2). Different from the important effect of channel turret Gln592 on toxin BeKm-1 binding [11], there was only about 13-fold drop in toxin BmKKx2 binding affinity when Gln592 residue was replaced by alanine (Figures 5G, 6B and Table 2). Since the alanine has shorter sidechain than that of cysteine, and the substitution of channel turret Gln592 by cysteine was found to have intermediate-impact on hERG channel function shown by the differential effects of mutant channel function in the presence or absence of reducing agent DTT [22]. In combination with the less effect of mutant hERG-Q592A on toxin BmKKx2 affinity (Figures 5G, 6B and Table 2), channel turret Gln592 did not play a critical role in scorpion toxin BeKm-1 or BmKKx2 binding since toxin BeKm-1 or BmKKx2 had the similar structures and common functional residues (Figure 7A and B).

Further comparison of the pharmacological data of toxin BmKKx2 and BeKm-1 towards mutant hERG channels showed a common feature that three turret regions including S5H, helix and HP played an unimportant role in toxin binding [10] (Figures 4, 5, and 6 and Table 2). In our previous work, the hERG channel turrets were predicted to possibly affect toxin BeKm-1 binding process because of its significant flexibility although they were likely far from the bound toxin BeKm-1 during the molecular dynamic simulations [10]. This inference was supported by about 10-fold drops of toxin BmKKx2 affinities towards mutant hERG-R582A, hERG-I583A, hERG-H587A, hERG-Q592A, hERG-Y597A, hERG-K608A (Figures 5, 6 and Table 2).

In summary, the comprehensive analysis of the pharmacological data of toxin BmKKx2 and BeKm-1 towards mutant hERG channels showed that channel turrets formed an open conformation in scorpion toxin binding [10] (Figures 5, 6 and Table 2). This open conformation was further shown in the toxin BmKKx2-hERG channel complex through the structural modeling using our previous toxin BeKm-1-hERG channel complex as the template [10] (Figure 7C). Such conformational feature of hERG channel turrets was also observed in the turrets of Kv1.2 channel bound by scorpion toxin maurotoxin [6] and the turrets of BKCa channel bound by scorpion toxin ChTX [8].

### Conserved function of hERG channel pore region in toxin BmKKx2 recognition

The pore regions of potassium channels play an important role during its interactions with the channel-blocking animal toxins [19,20]. In this work, the channel Ser631 was found essential for toxin BmKKx2 binding shown by about 104-fold drop of BmKKx2 affinity compared with that of wild-type hERG channel (Figures 5P, 6D and Table 2). As shown in Figure 4, the corresponding residues in the position of Ser631 near hERG channel selectivity filter were critical for toxin activities among different potassium channels [6,19,23], which also rationalized the conserved and critical function of Ser631 in the hERG channel pore region. In the previous cysteine-scanning experiments [11], the importance of Ser631 residue in hERG channel was less significant likely since its substitution by cysteine was found to have intermediate-impact on hERG channel function shown by the differential effects of mutant channel function in the presence or absence of reducing agent DTT [22]. As shown in Figure 7D, the polar and nonpolar interactions between channel Ser631 and toxin residues further illustrated the essential function of channel Ser631 for toxin binding in the modeled hERG channel-toxin BmKKx2 complex structure.

## Conclusions

In this work, we revisited the hERG potassium channel which has an unusual longer turret with the considerable conformational flexibility. With the help of a new specific scorpion toxin BmKKx2, the first specific blocker for hERG channels from scorpion *Mesobuthus martensii*, the turret role of hERG channel was re-investigated through the alanine-scanning mutagenesis. All pharmacological data indicated that different residue substitutions by alanine decreased the affinities of toxin BmKKx2 by about 10-fold compared with that of wild-type hERG channel, which revealed that channel turret played a secondary role in toxin binding. The further comprehensive analysis of the pharmacological data of scorpion toxins towards mutant hERG channels from the previous cysteine-scanning and present alanine-scanning mutagenesis showed that channel turrets formed an open conformation in scorpion toxin binding. Together, these findings were helpful to understand the unique role of hERG channel turrets in toxin recognition and enrich the diversity of turret structure-function relationships among the different potassium channels.

## Methods

### Clone, expression and purification of BmKKx2 peptide

The cDNA library of *Mesobuthus martensii* was constructed by our group as described previously [21]. cDNAs were cloned into pSPORT1 plasmids and transformed into *E. coli* DH5α cells (obtained from China Center for Type Culture Collection, CCTCC). Randomly chosen cDNA clones were sequenced to obtain a reliable representation of the toxin content in the venom gland.

After acquiring the full-length cDNA of BmKKx2, expression plasmid pGEX-6p-1-BmKKx2 was constructed using overlap PCR method. The primers used were: forward primer 1: 5’-CCTACAGATATTAAATGCAGTGCATCTTATCAATGT- TTCCCTGTTTG-3’; reverse primer 1: 5’-AACGTCCATTAGTCTTTCCGAAGCG-GCTTTTACAAACAGGGAAACATTG-3’; forward primer 2: 5’-GTGAATTC**GAT-GACGATGACAAG**CGTCCTACAGATATTAAATG-3’; reverse primer 2: 5’-TAGCTCGAGCTAGAAACAGTCGCATAAACCATTCACGCAACGTCCATTAG-3’. The restriction enzyme sites are underlined, and an enterokinase cleavage site is in bold. The PCR products were inserted into expression vector pGEX-6p-1 and sequenced with universal pGEX primers. *E. coli* Rosetta (DE3) cells were used to express BmKKx2 and its mutants according to previous techniques of our group [7,10,15,21].

### Alanine-scanning mutagenesis of hERG channel

The wild-type hERG-pEGFP-N2 vector was used in this work. Twenty-two mutants were chosen for study base on the structure analysis of hERG channel and our previous model [10]. All the channel mutants were constructed with the QuickChange site-directed mutagenesis kit (Stratagene), as described by the manufacture. The mutated expression plasmids were confirmed by sequencing after construction (Takara).

### Cell culture and transfection

Human embryonic kidney (HEK293) cells were cultured in Dulbecco's Modified Eagle's Medium (DMEM, Gibco) with 10% Fetal Calf Serum (Invitrogen Life Technologies, Carlesbad, CA), supplemented with 100 units/mL ampicillin and 100 μg/mL streptomycin in a humidified 5% CO_2_ incubator at 37°C.

Cells were transfected with 6 μg plasmid of wild-type or mutant hERG using the FuGene Transfection Method (Roche Diagnostics, Switzerland). Currents were recorded 24–48 hours after transfection in EGFP fluorescent cells at room temperature using the whole-cell patch-clamp mode with an EPC10 Amplifier (HEKA, Germany).

### Electrophysiology and data analysis

Cells expressing Kv channels were incubated in the external solution consisted of (in mM): 137 NaCl, 4 KCl, 1 MgCl_2_, 1.8 CaCl_2_, 10 D-Glucose and 10 HEPES (pH7.4 with NaOH); and the internal solution contained (in mM): 130 KCl, 1 MgCl_2_, 5 MgATP, 5 EGTA, 10 HEPES (pH 7.2 with KOH). For Kv1 channels and Kv4.2, currents were elicited by 200-ms or 500-ms depolarizing pulses from a holding potential of -80 to 50 mV, individually. While cells expressing hERG channel were depolarized from a holding potential of -80 mV to +40 mV for 500 ms then hyperpolarized to -120 mV for 1 s and current amplitudes were measured from the peak inward current at -120 mV.

For the recording of SKCa channels, the bath solution contained 130 mM sodium aspartate, 30 mM K^+^-aspartate, 2 mM CaCl_2_, 1 mM MgCl_2_, and 10 mM HEPES (pH 7.4 with NaOH). The pipette solution contained 145 mM K^+^-aspartate, 8.7 mM CaCl_2_, 2 mM MgCl_2_, 10 mM EGTA, and 10 mM HEPES (pH 7.2 with KOH) to achieve an intracellular free Ca^2+^ concentration of 1 μM. The membrane potentials were clamped to -120 mV for 50 ms (which was used for the current measurements), followed by a 400 ms voltage ramp from -120 to +60 mV and were kept for 5 s between ramps at -40 mV.

When toxin peptides were applied, 0.01% Bovine Serum Albumin (BSA) was added to the external solution to seal the tubes of the perfusion system. A multi-channel micro-perfusion system MPS-2 (INBIO Inc, Wuhan, China) was used to exchange the external recording bath solution. Current amplitudes were obtained using Pulse as acquisition software while data was analyzed with Sigmaplot 11.0 (SPSS Inc. USA). Dose response curves for the toxin concentration dependence of current inhibition were fitted with a modified Hill equation,

$$I_{\mathrm{toxin}}/I_{\mathrm{control}}=1/\left(1+\left[T\right]/{\mathrm{IC}}_{50}\right),$$

where IC_50_ is the half-maximum inhibition concentration, I is the peak tail current, [T] represents the concentration of toxins. Results are shown as mean ± S.E., and *n* is the number of experiments.

## Competing interests

The authors declare that they have no competing interests.

## Authors’ contributions

YTH, WXL and YLW designed the study. YTH and TL constructed the mutant channels. JJW was responsible for harvesting recombinated BmKKx2 peptide and its mutants. YTH carried out cell culture and transfection. YTH and JF performed electrophysiological experiments. YTH and JH analyzed data. YTH and YLW wrote the manuscript. All authors read and approved the final manuscript.

## Supplementary Material

Additional file 1: Figure S1 Circular dichroism spectra of wild-type BmKKx2 and mutants. (A) Circular dichroism spectra of recombinant BmKKx2, BmKKx2-R1A, BmKKx2-Y11A, BmKKx2-K23A and BmKKx2-F32A. (B) Circular dichroism spectra of BmKKx2, BmKKx2-K18A and BmKKx2-R20A. The measurement was carried out in the UV range of 250–190 nm at 25°C in water on a Jasco-810 spectropolarimeter with a concentration of 0.2–0.4 mg/mL. Click here for file
