# Supplementary figures and images for: Open conformation of hERG channel turrets revealed by a specific scorpion toxin BmKKx2
Source: Cell Biosci. 2014 Apr 11;4:18. doi: 10.1186/2045-3701-4-18 (PMC4108063; doi:10.1186/2045-3701-4-18)

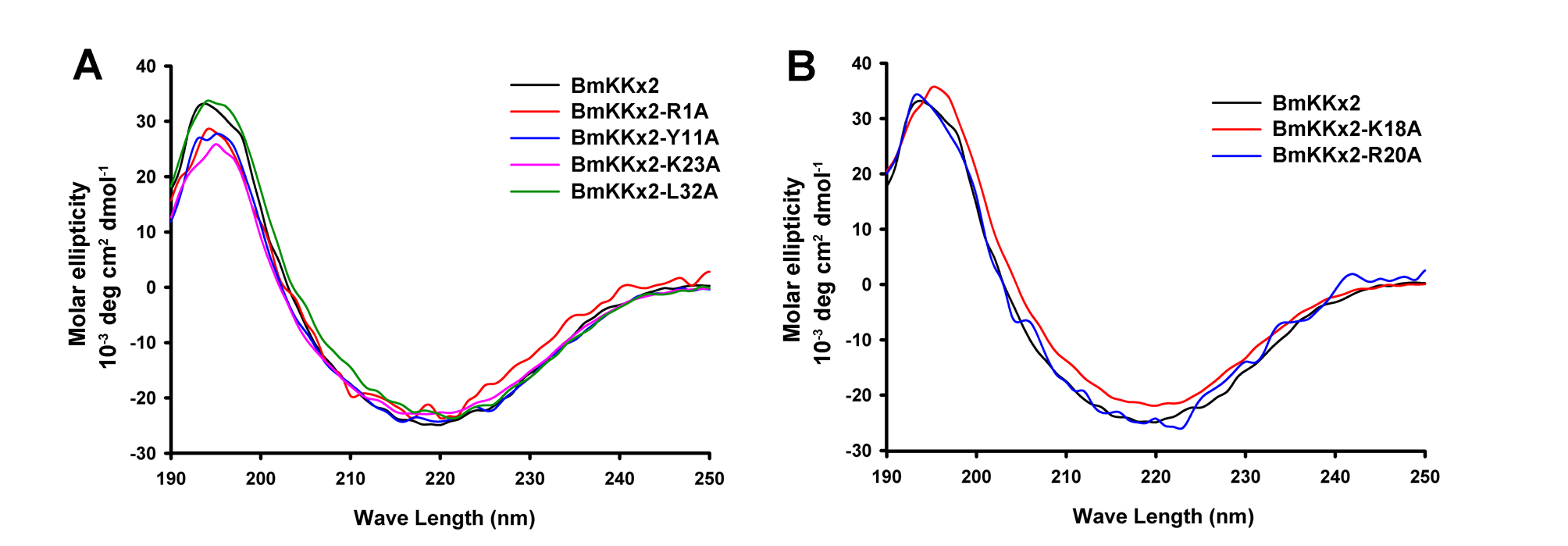

Supplement: Additional file 1: Figure S1 — Circular dichroism spectra of wild-type BmKKx2 and mutants. (A) Circular dichroism spectra of recombinant BmKKx2, BmKKx2-R1A, BmKKx2-Y11A, BmKKx2-K23A and BmKKx2-F32A. (B) Circular dichroism spectra of BmKKx2, BmKKx2-K18A and BmKKx2-R20A. The measurement was carried out in the UV range of 250–190 nm at 25°C in water on a Jasco-810 spectropolarimeter with a concentration of 0.2–0.4 mg/mL. [file 2045-3701-4-18-S1.tiff]
